# *Candidatus* Neoehrlichia mikurensis in Bank Voles, France

**DOI:** 10.3201/eid1812.120846

**Published:** 2012-12

**Authors:** Muriel Vayssier-Taussat, Danielle Le Rhun, Jean-Philippe Buffet, Narimane Maaoui, Maxime Galan, Emmanuel Guivier, Nathalie Charbonnel, Jean-François Cosson

**Affiliations:** Author affiliations: Institut National de la Recherche Agronomique, Maisons-Alfort, France (M. Vayssier-Taussat, D. Le Rhun, J.-P. Buffet, N. Maaoui);; Institut National de la Recherche Agronomique, Montferrier-sur-Lez, France (M. Galan, E. Guivier, N. Charbonnel, J.-F. Cosson)

**Keywords:** Candidatus Neoehrlichia mikurensis, rodents, bank vole, Myodes glareolus, France, zoonoses, wildlife, vector-borne infections, ticks, Ixodes ricinus, bacteria

## Abstract

To further assess the geographic occurrence, possible vectors, and prevalence of *Candidatus* Neoehrlichia mikurensis, we analyzed spleen tissues from 276 voles trapped close to human settlements in France; 5 were infected with the organism. Sequencing showed the isolates carried the same genotype as the bacteria that caused disease in humans and animals elsewhere in Europe.

Emerging infectious diseases substantially affect public health. Analysis of a database of 335 emerging infectious disease events indicated that 60.0% were zoonotic diseases, most (71.8%) of which originated in wildlife, and that zoonoses are increasing over time (*1*). Half of these emerging infection events involved bacteria belonging, for the most part, to the proteobacterial order *Rickettsiales* (*1*).

In Western Europe, the widespread and abundant *Ixodes ricinus* tick is the most common vector for human and animal pathogens and is also a major vector of pathogens responsible for rodent-borne diseases (*2*). Lyme borreliosis (Lyme disease), which is caused by infection with the bacteria *Borrelia burgdorferi* sensu lato, is the most prevalent tick-borne and rodent-borne illness. However, persons bitten by ticks can also be infected by other bacteria belonging to 3 main genera: *Anaplasma* spp., *Bartonella* spp. and *Rickettsiae* spp. Over the past 10 years, these bacterial species have been associated with tick-borne infections in humans (*2*). 

During the last decade, DNA of a new species of intracellular bacteria belonging to the family *Anaplasmataceae* was sequenced from isolates from ticks and rodents originating in Europe and Asia (*3*–*5*). The first isolate of this organism was obtained in 2004 from wild *Rattus norvegicus* rats and *I. ovatus* ticks from Japan (*6*). A comparison of the morphologic and molecular characterization of that isolate with earlier, closely related sequences in the GenBank database supported classification of the isolate in a novel genetic cluster within the family *Anaplasmataceae*; thus, the nomenclature “*Candidatus* Neoehrlichia mikurensis” was proposed for all current organisms in this group (*6*). 

Since the 2004 discovery of *Candidatus* N. mikurensis, the bacterium has been identified in different tick species, including *I. ricinus* ticks in Europe, and in small rodents (other than *R. norvegicus* rats) suspected of being the main reservoir for the bacterium (*7*–*10*). In 2010, human infection with C*andidatus* N. mikurensis was reported in a Swedish patient (*11*). In that same year, infections were described in 5 persons in Germany, Switzerland, and the Czech Republic (*12*). More recently, *Candidatus* N. mikurensis infection was reported in a dog in Germany (*13*). Signs and symptoms described in all cases were general and nonspecific (e.g., fever, cough, anemia, headache, pulmonary infiltrate, malaise, myalgia, joint pain, extreme fatigue, erythema), making diagnosis difficult, particularly in the absence of serologic tests. Thus, it is likely that the actual incidence of human *Candidatus* N. mikurensis infection in Europe is much higher than currently reported. 

Previous studies of *Candidatus* N. mikurensis in Europe have advocated for an assessment of the geographic occurrence, possible vectors, and prevalence of this microorganism. In this study we demonstrate the presence of *Candidatus* N. mikurensis in France, specifically in the bank vole (*Myodes glareolus*), a suspected reservoir for the microorganism.

## The Study

During the course of a 2008 study of the epidemiology of Puumala hantavirus, we trapped voles in the French Ardennes, a forested region on the border with Belgium, along a transect line of ≈80 km (*14*). Along this transect, we sampled 6 sites in forested areas and 4 sites in fragmented habitats (i.e., hedge networks). We euthanized the voles by cervical dislocation and determined their weight and sex. The animals were then dissected. Spleens were placed in RNA*later* Storage Solution (Sigma-Aldrich, St Louis, MO, USA) and stored at −20°C for further analyses. We used the DNeasy Blood and Tissue Kit (QIAGEN, Hilden, Germany) according to the manufacturer’s instructions to extract genomic DNA from a portion of the spleen into a final elution volume of 100 µL.

To test the spleen DNA for the presence of *Candidatus* N. mikurensis, we used PCR with specific primers targeting the *Candidatus* N. mikurensis *groEL* gene, as described (*13*): results were positive for 5 (1.8%) of 276 vole spleens tested. This prevalence lies within the range (0%–11.5%) found among small rodents trapped in other European countries (*7*,*9*). Rodents positive for *Candidatus* N. mikurensis were trapped in forested patches in Woiries (49.903°N, 4.763°E), Elan (49.655°N, 4.770°E), and Croix-aux-Bois (49.419°N, 4.824°E), France, and also in a hedge network in Cliron (49.802°N; 4.619°E), France, where the *I. ricinus* tick is also abundantly present (E. Ferquel, pers. comm.). All *Candidatus* N. mikurensis–positive bank voles were trapped in close proximity (300 m–1 km) to human settlements and villages.

All PCR products (a 1,024-bp fragment of the *groEL* gene) were sequenced in forward and reverse directions by Eurofins MWG Operon (Ebersberg, Germany). Sequences were aligned and analyzed for phylogenetic purposes by using SeaView version 4 (*15*). All sequences were identical and matched those of the *Candidatus* N. mikurensis *groEL* gene. Sequences showed the highest identity with gene sequences of *Candidatus* N. mikurensis isolates in Europe. In particular, the sequences showed 99% nt identity with sequences obtained for isolates from 2 humans (GenBank accession nos. EU810407.1 and EU810406.1) and a dog (GenBank accession no. EU432375) residing in Germany. The lowest sequence identity was shared with isolates obtained outside of Europe: 95% nt identity with isolates from wild rodents in the People’s Republic of China (GenBank accession nos. JQ359066, JQ359067, and JQ359068); 94% identity with isolates from wild rodents and *I. ovatus* ticks in Japan (GenBank accession nos. AB074461 and AB084583, respectively); and 91% identity with isolates from raccoons in the United States (GenBank accession no. EF633745).

We constructed the phylogenetic tree by using the neighbor-joining method and a Kimura 2-parameter distance. Bootstrap analysis was performed on 1,000 replicates.

Phylogenetic analysis of the comparatively long *groEL* gene sequence showed highly significant clustering of the samples into 3 groups supported by high bootstrap values (Figure), suggesting the existence of at least 3 types of *Candidatus* N. mikurensis sequence variants. Clustering was not related to the host or to the geographic origin of the sample. All sequences obtained from *Candidatus* N. mikurensis isolates from bank voles in France clustered together with sequences that had been amplified from samples from sick humans or animals in Europe.

**Figure:**
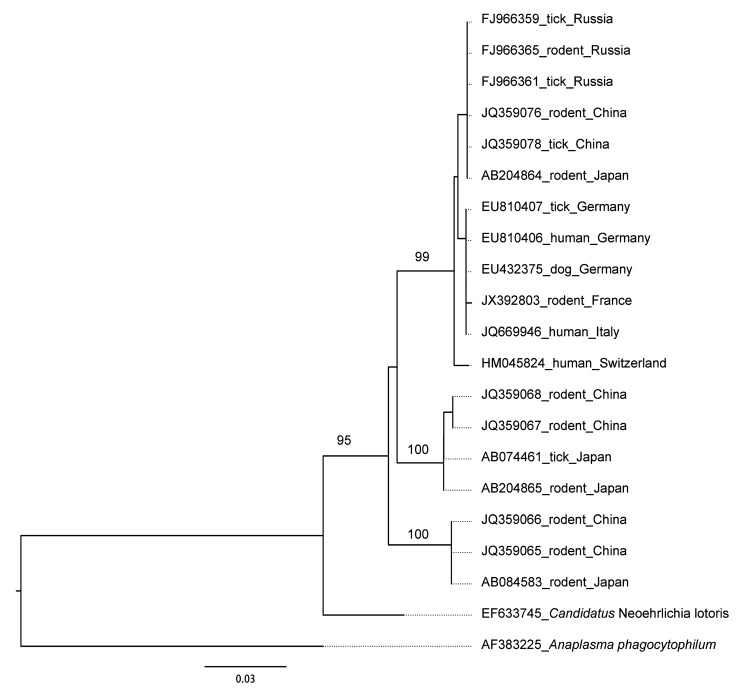
Phylogenetic relationships, as determined on the basis of the sequence of the *groEL* gene, between the unique *Candidatus* Neoehrlichia mikurensis genotype detected in a population of bank voles from the French Ardennes and other *Candidatus* N. mikurensis genotypes from other geographic regions. The phylogenetic tree was constructed by using the neighbor-joining method with the Kimura 2-parameter distance model. Bootstrap analysis was performed on 1,000 replicates; values are indicated at the nodes. GenBank accession numbers are indicated for each sequence used. The *groEL* sequence of *Anaplasma phagocytophilum* (accession no. AF383225) was chosen as an outgroup in the phylogenetic tree. Scale bar indicates estimated evolutionary distance.

## Conclusions

Our results show that *Candidatus* N. mikurensis is present in bank voles in France; furthermore, the bacteria carried by the rodents in this study were of the same genotype as the *Candidatus* N. mikurensis that caused disease in humans and animals elsewhere in Europe. These findings have implications for public health because small rodents are the main source of blood meals for the larvae and nymphs of *I. ricinus* ticks and because the *Candidatus* N. mikurensis–positive rodents in our study were collected in close proximity to human dwellings. Thus, we are currently collecting *I. ricinus* ticks in the same region of France where we trapped the bank voles to determine whether ticks are a possible vector and a source of transmission of *Candidatus* N. mikurensis to humans.

In addition, nephropatia epidemica, a hemorrhagic fever caused by Puumala hantavirus, is endemic in the geographic region from which the bank voles in our study were collected. The symptoms for nephropatia epidemica (body aches, chills, sweats, fatigue, and high fever) are mild and could be confused with those caused by infection with *Candidatus* N. mikurensis. Diagnosis of *Candidatus* N. mikurensis infection relies only on PCR amplification of the bacterial DNA. The absence of serologic tests for *Candidatus* N. mikurensis, combined with the lack of knowledge relating to these bacteria among medical practitioners, makes diagnosis of *Candidatus* N. mikurensis infection particularly difficult. Thorough surveillance and improved diagnostic tools are required to gain more insight into the relevance of *Candidatus* N. mikurensis to public health.
